# *Coxiella burnetii* Delays Apoptosis and Causes Lytic Cell Death in Human Neutrophils In Vitro

**DOI:** 10.3390/pathogens15090929

**Published:** 2026-09-03

**Authors:** Kannan Thangamani, Yan Zhang, Guoquan Zhang

**Affiliations:** Department of Microbiology and Molecular Immunology, University of Texas at San Antonio, San Antonio, TX 78249, USA; k.thangamani@northeastern.edu (K.T.); yan.zhang@utsa.edu (Y.Z.)

**Keywords:** *Coxiella burnetii*, Nine Mile phase II, human neutrophil, apoptosis, autophagy, neutrophil extracellular traps

## Abstract

*Coxiella burnetii* is an obligate intracellular Gram-negative bacterium that causes acute and chronic Q fever in humans. Neutrophils are considered the major innate cells with a primary role in controlling extracellular bacteria. Despite the obligate intracellular lifestyle of *C. burnetii*, our previous study demonstrated that neutrophils are crucial for host defense against *C. burnetii* infection in mice. Our recent study also showed that virulent *C. burnetii* Nine Mile phase I (NMI) and avirulent NM phase II (NMII) strains differentially modulate autophagy and apoptosis in murine neutrophils. However, the interaction between human neutrophils and *Coxiella* remains unexplored. In this study, we investigated how human neutrophils interact with *C. burnetii*. The results indicated that NMII bacteria were able to infect and reside in autophagolysosomal compartments of human neutrophils. Additionally, NMII bacteria inhibited the expression of ANNEXIN-V and the activation of caspase-3, resulting in delayed apoptosis in neutrophils. Live imaging showed that NMII bacteria actively swarm and recruit human neutrophils to form localized neutrophil clusters, which deploy neutrophil extracellular traps (NETs) to restrain bacteria. The deployment of slow-diffusing NETs in localized clusters was observed with NMII-infected human neutrophils in a dose-dependent manner. Furthermore, the observation of *Coxiella* colocalization with the deployed NETs demonstrates the ability of human neutrophils to immobilize and contain *C. burnetii.* We also found that the neutrophil’s decision to undergo constitutive apoptosis or NETosis depends on the activation of the PI3K-autophagic pathway. Collectively, this study provides novel information for understanding the mechanism of human neutrophil interaction with *Coxiella*.

## 1. Introduction

*Coxiella burnetii* is an obligate intracellular Gram-negative bacterium that causes the worldwide zoonotic disease, Q fever. Human Q fever is mainly transmitted via the respiratory route by inhalation of *C. burnetii*-contaminated aerosols. The disease commonly manifests as flu-like symptoms, but occasionally chronic infection occurs, leading to life-threatening endocarditis [1]. The innate immune cells, including neutrophils, macrophages and dendritic cells, play crucial roles in establishing a robust host immune defense against respiratory bacterial infections [2,3]. The functions of neutrophils in controlling bacterial dissemination and the disease progression have been demonstrated by neutrophil depletion experiments against several bacterial pathogens, including *Streptococcus pneumoniae*, *Legionella pneumophila* [4], *Klebsiella pneumoniae* [5] and *C. burnetii* [6]. The abilities of neutrophils to induce pro-inflammatory responses, phagocytosis and bactericidal via reactive oxygen species, antimicrobial proteins, and serine proteases make them indispensable for host defense against bacterial pathogens. Additionally, neutrophils have extracellular responses like secretion of granule proteins and formation of extracellular traps to trap and kill bacteria. However, dysregulated immune response during bacterial infections could lead to an accumulation of neutrophils and heightened inflammation, resulting in bacterial pneumonia [7,8]. Uncontrolled bacterial pneumonia can escalate into respiratory distress syndrome (ARDS) and potentially fatal disease [8].

Our previous study [6] indicated that *C. burnetii* virulent Nine Mile phase I (NMI) infection induced more severe clinical disease, bacterial burden and histopathological changes in spleen and lungs in neutrophil-depleted mice than wild-type control mice, suggesting that neutrophils are required for host defense against virulent *C. burnetii* infection in mice. In addition, infecting immunocompetent BALB/c mice with NMI *C. burnetii* resulted in a dysregulated immune response, evident from the accumulation of neutrophils in the lungs, spleen, and liver [9]. These observations provide substantial evidence of systemic host–pathogen interactions involving neutrophils and *C. burnetii*, thus warranting a closer examination of this relationship.

**Figure 1:**
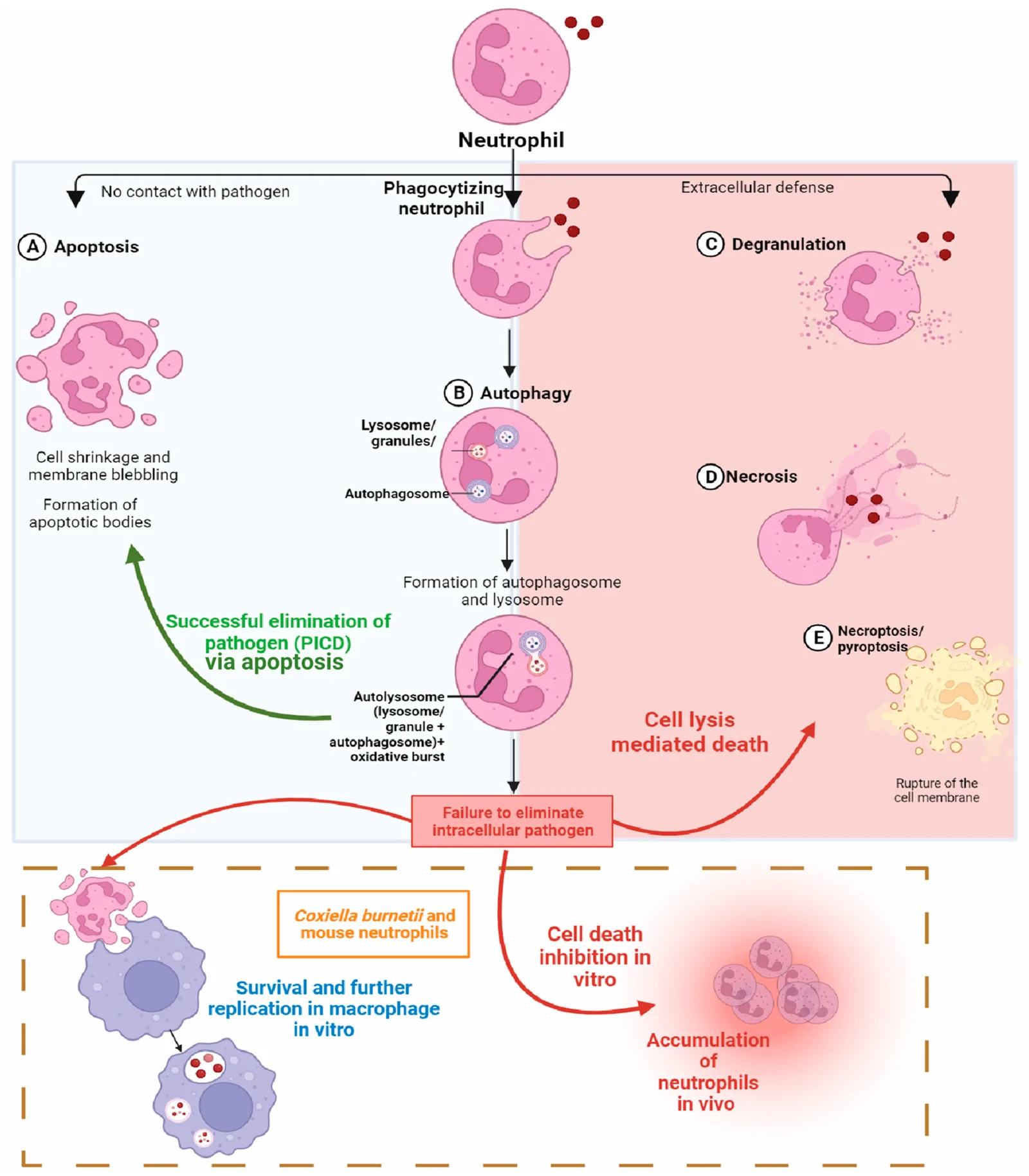
Neutrophil death is intertwined with its immune response. Neutrophils that encounter bacteria can either undergo apoptosis—a non-inflammatory route of pathogen-induced cell death—or a pro-inflammatory cell lysis-mediated death called necrosis. The balance between these two mechanisms during a bacterial infection plays a pivotal role in the inflammatory response, pathogen clearance, and disease progression. *Coxiella* is an obligate intracellular pathogen known to subvert the mouse neutrophil response. By studying how neutrophils die, we can infer the immune response against the pathogen. Created in BioRender. Thangamani, K. (2026) https://BioRender.com/.

Subsequent in vitro studies from our lab have elucidated the capability of *C. burnetii* to infect bone marrow-derived mouse neutrophils. Notably, the infection rate of *Coxiella* in these mouse neutrophils was lower compared to macrophages, reinforcing macrophages as the bacterium’s preferred host [10]. Moreover, co-culture experiments have demonstrated that *C. burnetii*, despite being unable to replicate, can remain viable within mouse neutrophils. When these neutrophils were subsequently phagocytized by macrophages, *C. burnetii* was able to resume its replication [10]. This observation indicates a complex survival strategy of the bacterium, leveraging different cell types within the immune system for its replication and persistence. Our previous studies also demonstrated the ability of *C. burnetii* to subvert immune defenses of mouse bone marrow-derived neutrophils as well as modulate their central functions like apoptosis and autophagy and thus their survival (Figure 1). In addition, although it has been reported that upon *C. burnetii* infection, human neutrophils exhibit a reduced rate of infection and inhibition of the NADPH-dependent oxidative burst mechanism [11], the mechanism of human neutrophil interaction with *C. burnetii* remains unexplored.

In this study, primary human neutrophils were isolated from human blood and used to investigate how human neutrophils interact with *C. burnetii* NMII bacteria. The results indicated that NMII bacteria were able to infect and reside in autophagolysosomal compartments of human neutrophils. Additionally, NMII bacteria inhibited the expression of Annexin V and the activation of caspase-3, which resulted in delayed apoptosis in NMII-infected neutrophils. We also found that NMII bacteria actively swarm and recruit human neutrophils to form localized neutrophil clusters that deploy NETs to trap bacteria. Thus, this study provides novel insights into the mechanisms underlying the interaction between human neutrophils and *C. burnetii*.

## 2. Materials and Methods

### 2.1. Blood Samples for Isolation of Human Neutrophils

Buffy coat (enriched leukocyte fraction) samples were obtained from the South Texas Blood and Tissue Center, derived from whole blood donated on the same day. These buffy coats were collected immediately post-extraction and were continuously stored on ice until neutrophil isolation on the same day. The study implemented specific exclusion criteria for donor selection: only individuals aged 18 to 40 years were eligible to participate, in line with findings from previous research indicating compromised phagocytic killing capabilities in neutrophils from older individuals compared to those in young and middle-aged cohorts [12]. Moreover, to exclude the influence of infection-associated neutrophilia, extensive screening for antibodies against a range of viral and bacterial infections was conducted for all potential donors. Inclusion in the study was restricted to those donors whose test results were negative.

### 2.2. Neutrophil Isolation

Prior to neutrophil isolation, the red blood cells (RBCs) in the buffy coat were removed using EasySep™ RBC Depletion Reagent (Catalog # 18170) (STEMCELL Technologies, Vancouver, BC, Canada). The sample was prepared by mixing equal volumes of buffy coat to recommended medium within a volume range of 1 to 5 mL. Ethylenediaminetetraacetic acid (EDTA) was added to achieve a final concentration of 6 mM. The 3 mL sample was diluted with an equal volume (3 mL) of the recommended medium (RPMI supplemented with 10% FBS). The depletion reagent was vortexed for 30 s to ensure uniform dispersion. Next, 150 µL of this reagent (50 µL/mL of the original sample volume) was added to the sample, and the procedure was immediately followed by the next step without incubation. The uncapped tub was placed into the magnet and incubated at room temperature for 5 min. The cell suspension was then carefully pipetted from a 14 mL tube using a 10 mL pipette into a new tube, ensuring that the entire clear fraction was collected from top to bottom. For optimal recovery, a small volume of RBCs (up to 10% of the starting sample volume) was also collected. The sequential steps of adding 150 µL of depletion reagent, incubating in the magnet for 5 min, and transferring the suspension to a new tube were repeated once. For a third and final magnetic separation, this suspension was incubated in the magnet for 5 min without adding further depletion reagent. After this final incubation, the cell suspension was carefully pipetted into a new tube. At this stage, the isolated cells were ready for downstream neutrophil isolation.

Human neutrophils were isolated from buffy coat acquired from the South Texas Blood and Tissue Center using the EasySep™ Human Neutrophil Isolation Kit (STEMCELL Technologies, Vancouver, BC, Canada #17957). The RBC-depleted buffy coat was adjusted to a cell concentration of up to 5 × 10^7^ cells/mL. A 2 mL sample was transferred into a 14 mL (17 mm × 95 mm) polystyrene round-bottom tube. The isolation Cocktail was added to the sample at 50 µL/mL, mixed, and incubated at 2–8 °C for 5 min. Subsequently, 80 µL of RapidSpheres™ (STEMCELL Technologies, Vancouver, BC, Canada). (40 µL/mL of sample), vortexed for 30 s prior to addition, were mixed and incubated at 2–8 °C for 3 min. RPMI medium was added to top up the sample to a final volume of 5 mL. The tube (without the cap) was placed into the EasySep™ magnet (STEMCELL Technologies, Vancouver, BC, Canada). and incubated at room temperature for 5 min. The enriched cell suspension was carefully pipetted into a new 14 mL tube. To ensure the complete removal of residual cells and unbound RapidSpheres, this new tube containing the isolated cells was placed into the magnet for an additional 5 min without the addition of further antibodies.

### 2.3. C. burnetii Strain and Growth Conditions

*C. burnetii* Nine Mile phase II (NMII) clone 4 (RSA 439) was used in this study. This avirulent strain was originally derived from the virulent Nine Mile phase I strain [13]. NMII bacteria were grown in acidified citrate cysteine medium 2 (ACCM-2) [14]. Bacteria were pelleted by centrifuging at 15,000× *g* for 30 min. The supernatant was discarded, and the bacteria were washed twice with sterile 1× phosphate-buffered saline (PBS). The bacterial load in the culture stock was determined by analyzing the *com1* gene copy number from the genomic DNA through quantitative real-time PCR (qRT-PCR) [15]. The *com1* gene encodes a 27 kDa protein conserved among various strains of *C. burnetii* across different clinical and geographical sources.

Genomic DNA was isolated from 10 µL of bacterial culture by incubating for 18 h at 60 °C in lysis buffer (composed of 1 M Tris, 0.5 M EDTA, 7 mg/mL glucose, 28 mg/mL lysozyme) with 10 µL of proteinase K (20 mg/mL). Additionally, 21 µL of 10% SDS was added, followed by incubation for 1 h at room temperature. The DNA was extracted using the High Pure PCR Template Preparation Kit (Roche, Indianapolis, IN, USA), according to the manufacturer’s guidelines. SYBR green-based qPCR analysis (Applied Biosystems, Foster City, CA, USA) was performed using *com1* gene-specific primers, and the absolute bacterial count was determined using the standard curve generated using recombinant plasmid DNA (*com1* gene ligated into pBluescript vector (Thermo Fisher Scientific, Carlsbad, CA, USA)). Counted cells were aliquoted and stored at −80 °C until use.

### 2.4. C. burnetii Infection of Human Neutrophils

The isolated neutrophils were counted and plated at a density ranging from 5 × 10^5^ to 1 × 10^6^ cells per well in 24-well non-treated cell culture plates. The culture medium consisted of RPMI 1640 (Life Technologies, Carlsbad, CA, USA) supplemented with 10% (*v*/*v*) heat-inactivated fetal bovine serum (FBS; Gibco, Grand Island, NY, USA) and 20 mM HEPES buffer (hereafter referred to as RPMI 10%). Cells were acclimatized at 37 °C with 5% CO_2_ for 30 min before infection with NMII at multiplicities of infection (MOI) of 10 and 100. Following infection, the cells were maintained in the incubator until the designated time points of 12, 24, 36, or 48 h, depending on the experiment.

### 2.5. Detecting C. burnetii Intracellular Infection by ImageStream Cytometry

Imaging flow cytometry integrates the rapid sampling capability of traditional flow cytometry with the additional benefit of capturing images of each cell. This combination offers a richer dataset, providing not only total fluorescence intensity from each channel but also spatial information about the cells. The ImageStream system v6.3 (developed by Luminex) employs CCD (charge-coupled device) cameras to acquire multiple images of each cell, including brightfield, darkfield, and various fluorescent channels. Furthermore, more complex metrics like correlation, texture, and granularity can be assessed, providing insights into marker localization and cell morphology [16].

Isolated neutrophils were infected with *C. burnetii* at MOIs of 10 and 100 for 24 h. LysoTracker™ Blue DND-22 (Invitrogen, Thermo Fisher Scientific, Carlsbad, CA, USA #L7525), a live-cell stain that specifically targets acidic organelles, was added to the cells at a final concentration of 75 nM during the last hour of incubation. At the 24 h endpoint, the cells were collected by centrifugation at 300× *g* for 5 min. The cells were washed twice with sterile 1× PBS to remove extracellular bacteria and media, fixed with 2% formaldehyde, and permeabilized with ice-cold methanol for 10 min. This permeabilization step is necessary for the antibody to reach intracellular vacuoles containing *C. burnetii*. Identification of *C. burnetii* was performed using a rabbit antibody raised against a 28 kDa protein (P28), which is immunodominant in *C. burnetii* isolates originating from milk, ticks, and humans with acute Q-fever [17]. This protein has been used in detection of acute Q-fever. From here on this will be referred to as anti-P28 or anti-*Coxiella* antibody. Permeabilized cells were stained with the rabbit anti-P28 primary antibody for 3 h at room temperature. Following centrifugation at 100× *g* for 2 min, the cells were washed twice before incubation with a goat anti-rabbit PE secondary antibody for 1 h. The cells were finally washed again, resuspended in 100 µL of 1× PBS, and passed through a 70 µm filter to prevent clogging of the cytometer.

### 2.6. Detecting C. burnetii Intracellular Infection by Immunofluorescence Staining

Isolated neutrophils were infected with *C. burnetii* at an MOI of 10 and 100 and plated on coverslips at a concentration of 1 × 10^6^ cells/mL in RPMI supplemented with 10% FBS. The coverslips were pre-coated with poly-L-lysine (Sigma-Aldrich, St. Louis, MO, USA P4707) for 1 h at 37 °C, followed by rinsing with sterile water to remove excess poly-L-lysine. After 12 and 24 h, the coverslips were washed gently twice with 1 mL of sterile 1× PBS using a pipette, taking care not to detach the neutrophils. The cells were then fixed with 2% paraformaldehyde (PFA) and permeabilized in ice-cold methanol for 10 min (followed by a 1× PBS wash) and then blocked with 2% normal goat serum (Gibco) for 1 h at room temperature. After washing with sterile 1× PBS, the coverslips were incubated overnight with rabbit anti-P28 at 4 °C and counterstained with goat anti-rabbit IgG (H + L) cross-adsorbed PE-conjugated secondary antibody and anti-human MPO FITC (BioLegend, San Diego, CA, USA) for 1 h at room temperature. The cells were then stained with 20 µM Hoechst 33342 for 5 min and mounted on slides using ProLong Gold Antifade Mountant (Invitrogen, Thermo Fisher Scientific, Carlsbad, CA, USA). Z-stack images of different regions of the coverslips were captured using a Zeiss 710 NLO 2P (Carl Zeiss Microscopy GmbH, Jena, Germany) confocal microscope at 10×, 20×, 40×, and 63× magnification. Permeabilized cells were stained with the rabbit anti-P28 primary antibody for 3 h at room temperature. Following centrifugation at 100× *g* for 2 min, the cells were washed twice before incubation with a goat anti-rabbit PE secondary antibody for 1 h. The cells were finally washed again, resuspended in 100 µL of 1× PBS, and passed through a 70 µm filter to prevent clogging of the cytometer.

### 2.7. Measuring Phosphatidylserine Expression by Annexin-V Staining

Neutrophils were collected at designated time points and washed three times with 1× PBS. Phosphatidylserine externalization was determined via flow cytometry using an Annexin V-Ready conjugate (Invitrogen, Thermo Fisher Scientific, Carlsbad, CA, USA) according to the manufacturer’s instructions. Propidium iodide (PI) staining was used to distinguish early apoptotic cells from late apoptotic or necrotic neutrophils. The cells were analyzed using a CytoFLEX (Beckman Coulter, Brea, CA, USA) or BD LSR-II flow cytometer. Thirty thousand events were collected for each sample. Data were analyzed using CytExpert v2.5 (CytoFLEX) and FlowJo software v10.9 (Tree Star, Inc., Ashland, OR, USA) after compensation with single-stain controls.

### 2.8. Fluorescent Western Blot for Detection of Apoptosis and Autophagy-Related Proteins

A total of 1 × 10^6^ neutrophils were seeded in a 6-well plate in RPMI medium supplemented with 10% FBS. After 12 and 24 h of infection with *C. burnetii* at MOIs of 10 and 100, neutrophils were collected by gentle agitation via pipetting and washed with cold 1× PBS to remove the bacteria and medium. Cell pellets were lysed with 100 µL of ice-cold RIPA lysis buffer (Thermo Fisher Scientific, Waltham, MA, USA) supplemented with phosphatase and protease inhibitors (Invitrogen, Carlsbad, CA, USA) for 15 min on ice. Protein lysates were separated from cellular debris by centrifuging at 14,000× *g* for 15 min. Extracted protein was quantified using the Pierce BCA Protein Assay Kit (Thermo Scientific, Waltham, MA, USA). The samples were diluted in 2× Laemmli buffer (Bio-Rad Laboratories, Hercules, CA, USA) containing β-mercaptoethanol (Sigma-Aldrich, St. Louis, MO, USA) and heated at 95 °C for 10 min. A total of 15 µg of protein was separated on a 4–20% Tris-glycine gel by electrophoresis and transferred onto a nitrocellulose membrane. After blocking with a low-fluorescence blocking buffer for 1 h at room temperature, the membranes were probed with a mouse anti-LC3B (BioLegend, San Diego, CA, USA) autophagy marker and rabbit anti-cleaved caspase-3 (Cell Signaling Technology, Danvers, MA, USA). They were then counter-labeled with anti-rabbit Alexa Fluor 680 and anti-mouse Alexa Fluor 800 (Thermo Fisher Scientific, Waltham, MA, USA). Anti-GAPDH 555 (Invitrogen, Carlsbad, CA, USA) was used for loading control detection. Blots were imaged using theiBright FL-1500 imaging system (Thermo Fisher Scientific, Waltham, MA, USA) at the corresponding excitation and emission wavelengths of the secondary antibodies and target proteins. iBright Analysis software v5.2 (Thermo Fisher Scientific, Waltham, MA, USA) was used to further isolate, quantify, and normalize the protein bands of interest.

### 2.9. Microscopic Live Imaging NET Assay

Neutrophils were incubated in phenol red-free RPMI medium supplemented with 10% FBS, containing 20 µM Hoechst 33342 and 5 nM SYTOX Green Nucleic Acid Stain (Invitrogen), in a clear, flat-bottom 96-well plate at a density of 2 × 10^5^ cells per well. After 30 min, neutrophils were challenged with *C. burnetii* at multiplicities of infection (MOI) of 10 and 100. For the subsequent 240 min, images were captured using an EVOS M5000 Live-Cell Imaging System equipped with an on-stage incubator maintaining 37 °C and 5% CO_2_. A set of two images—Hoechst (excitation/emission: 350/461 nm) and SYTOX Green (excitation/emission: 504/523 nm)—was acquired every 3 min at 10×) magnification across four distinct fields per well.

### 2.10. Quantification of NETs

Individual NET release was quantified using EVOS cell analysis software v 1.5.1500.493 (Thermo Fisher Scientific, Waltham, MA, USA) by filtering for size and circularity to exclude smaller, circular apoptotic cells. The percentage of NET production was calculated relative to the number of Hoechst-stained nuclei at time zero. Conversely, the percentage of non-NET apoptotic SYTOX Green-positive cells was calculated by filtering for size and circularity to exclude non-spherical, larger NETs. Images from different fields at the same time point were quantified simultaneously using the software’s (v1.5.1500.493) Batch Run tool. Random manual verification was subsequently performed by reviewing the images after automated software detection. Additionally, group NET release—characterized by multiple neutrophils expanding over an area—was measured using ImageJ software v1.54f (National Institutes of Health (NIH), Bethesda, MD, USA).

### 2.11. Statistical Analysis

At least three independent experiments were conducted for all data presented, utilizing neutrophils from at least three different healthy volunteers. Statistical analyses were performed using GraphPad Prism v9.5.1 (GraphPad Software, San Diego, CA, USA) Statistical analysis involved the examination of differences between experimental groups using Student’s *t*-test and ANOVA. Depending on the experiment, repeated measures (RM) one-way ANOVA with Tukey’s multiple comparison test (fixed time point, variable infection or vice versa) or ordinary two-way ANOVA (multiple timepoints and infection groups) followed by Dunnett’s multiple comparison test were used. The figure legends contain the specific statistical analysis for each experiment.

## 3. Results

### 3.1. Colocalization of C. burnetii in Lysosomal Compartments of Human Neutrophils

When neutrophils engulf a pathogen into a phagosome, an essential step in pathogen destruction involves the fusion of antimicrobial granules into the compartment. In macrophages, this process relies on lysosomes, which create a highly acidic environment with a low pH to kill and digest invaders. Notably, *C. burnetii* is adapted to thrive and replicate within these acidic compartments, particularly inside macrophages [18].

**Figure 2:**
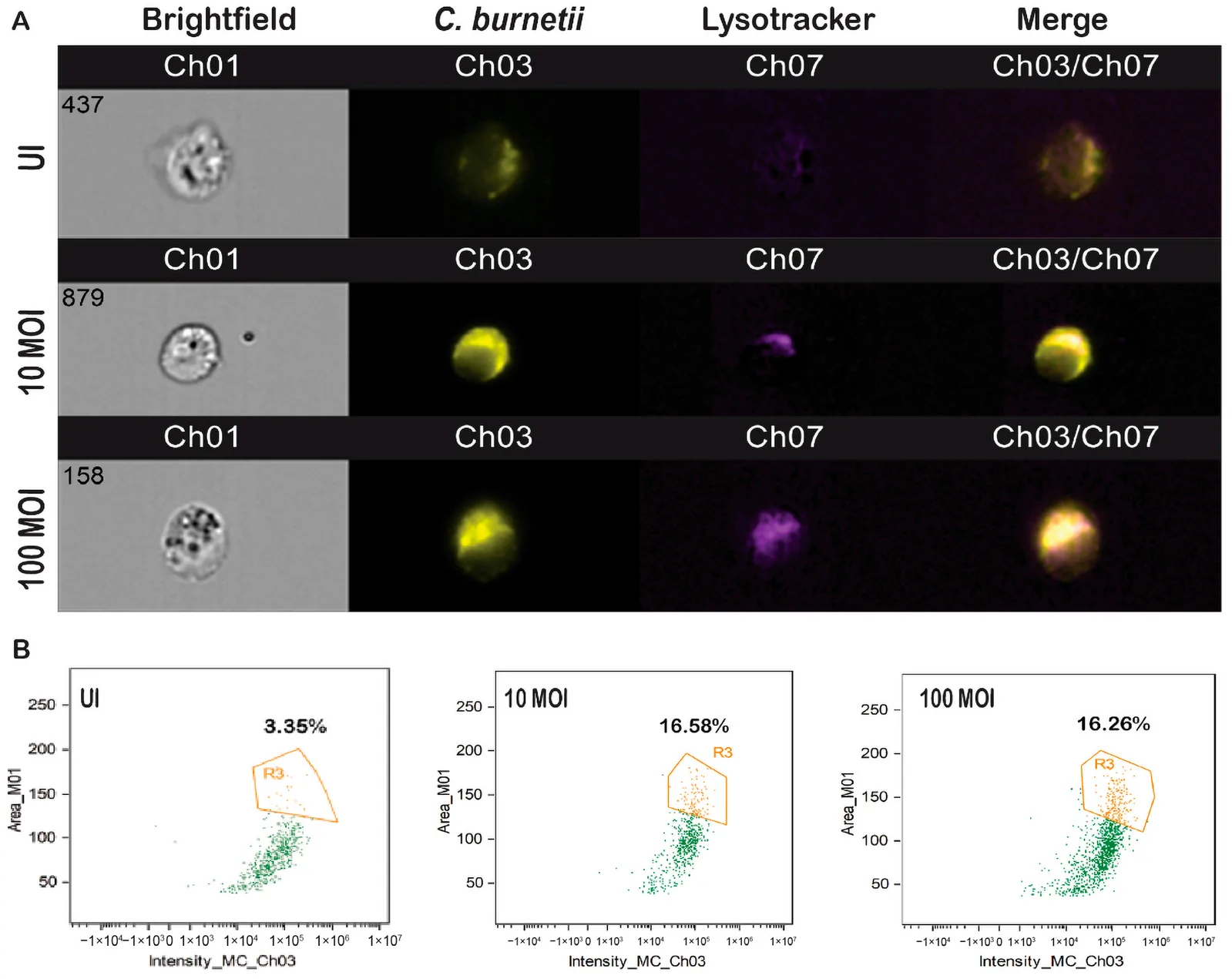
Intracellular infection of *C. burnetii* in human neutrophils I. (**A**) Representative images of human neutrophils from UI (uninfected), 10 MOI and 100 MOI groups, as brightfield and immunostained for *C. burnetii* (yellow) and LysoTracker (purple) and Merge. Magnification: 60×. (**B**) Representative FACS plots showing population double-positive for intensity of Lysotracker (*y*-axis) and intensity of anti-P28 (*C. burnetii*).

To confirm the intracellular infection of *C. burnetii* in human neutrophils, we employed LysoTracker, a live-cell stain that specifically targets acidic organelles. Given the non-adherent nature of human neutrophils, we utilized imaging flow cytometry, which enables simultaneous analysis of fluorescently tagged cells through both microscopy and flow cytometry. As shown in Figure 2A, the images progress from left to right, showing brightfield, anti-*Coxiella* staining in yellow, and lysosomes marked by LysoTracker in purple. Neutrophils infected at an MOI of 10 and 100 demonstrated a positive signal for anti-*Coxiella* staining (yellow) along with increased lysosomal activity (blue). As shown in Figure 2B, to determine the infection rates, a region was delineated to select for cells in focus (termed “R2”), which were then gated for single cells (named “R3”). These single cells were further gated for a population double-positive for the presence of LysoTracker and intracellular bacteria (yellow), labeled “R4”. The infection rate was determined to be ≈17% for 10 MOI and ≈16% for 100 MOI. Thus, the infection rates are similar between MOI of 10 and 100 NMII-infected human neutrophils. 

**Figure 3:**
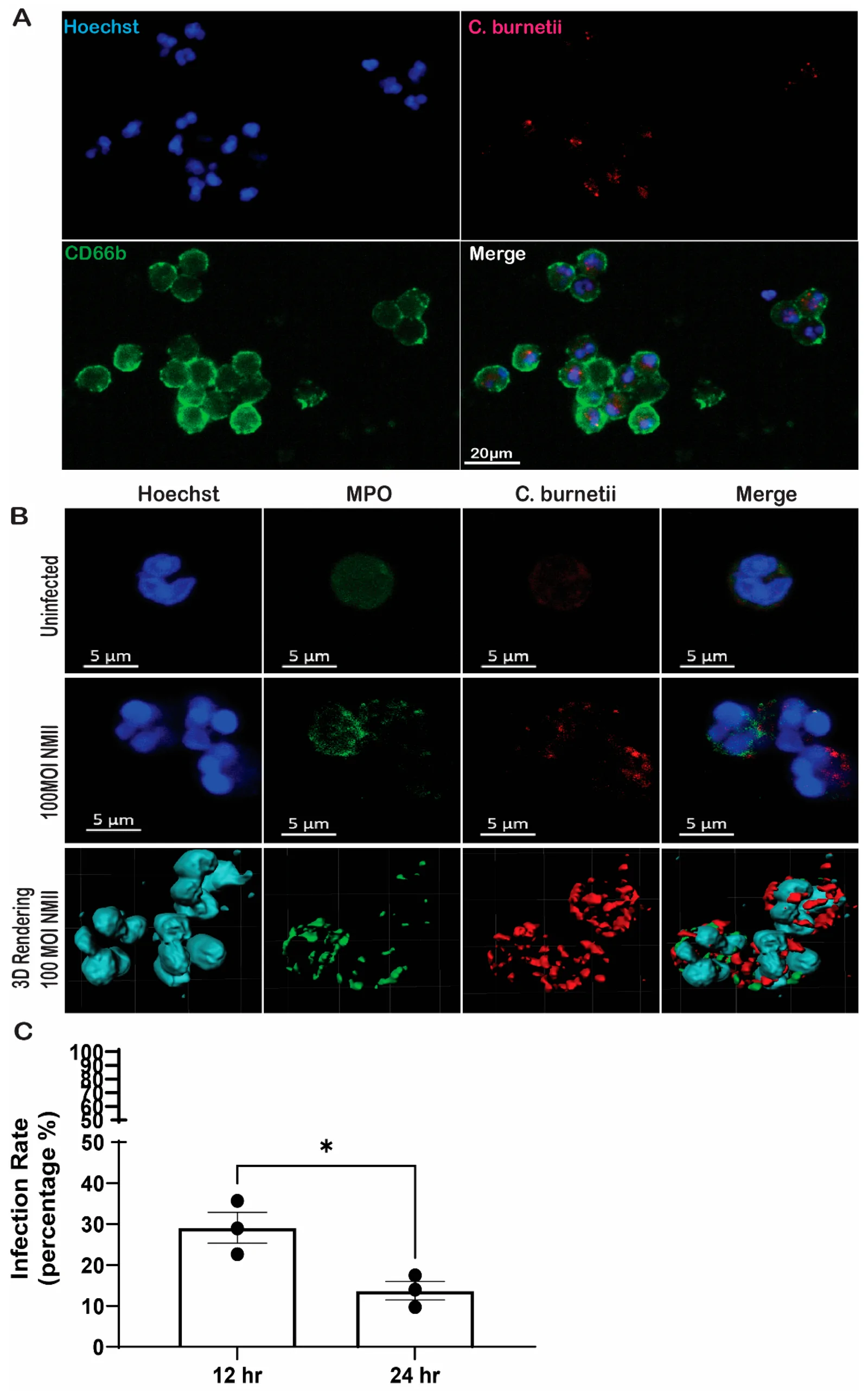
Intracellular infection of *C. burnetii* in human neutrophils. Purified neutrophils were infected with an MOI of 100 of *C. burnetii* for 12 and 24 h. (**A**) Representative images of human neutrophils from uninfected (UI) and 100 MOI groups, immunostained for *C. burnetii* (red), DNA (blue), and CD66b (green), alongside the merged image. Scale bars: 20 µm. (**B**) Images of human neutrophils from UI and 100 MOI groups, immunostained for anti-human myeloperoxidase conjugated with FITC (green), *C. burnetii* (red), and DNA (blue). Infection of neutrophils by *C. burnetii* was imaged using a Zeiss confocal microscope. Scale bars: 5 µm. A 3D model of the infected neutrophil was rendered using IMARIS software showing *C. burnetii* (red), DNA (blue), CD66b (green), and merge. (**C**) The bar graph represents the infection percentage from 100 MOI groups at 12 and 24 h time points. Data are mean ± SEM for *n* = 3. Each dot represents an independent trial. * *p* < 0.05 by student *t*-test. Magnification: 60×.

In addition, the intracellular localization of *C. burnetii* in human neutrophils was analyzed by confocal microscopy after immunofluorescence staining. As shown in Figure 3A, the localization of *C. burnetii* adjacent to the nucleus, stained by Hoechst (blue) and enclosed by CD 66b, confirms the presence of intracellular infection. To validate intracellular infection during delayed apoptosis, we examined two different time points by infecting cells at a MOI of 100 for 12 and 24 h. As shown in Figure 3B, the panels display the nucleus (blue), *C. burnetii* (red), and myeloperoxidase (MPO; green). At 100 MOI, *C. burnetii* was present within the cells but did not colocalize with MPO. Furthermore, IMARIS software v9.9 (Oxford Instruments/Bitplane, Zurich, Switzerland) was used to generate 3D models of the infected cells. Figure 3C presents the infection rates, calculated as the percentage of *C. burnetii*-positive cells relative to the total number of Hoechst-stained nuclei. An increase in infection duration led to a decrease in the infection rate, dropping from ≈30% at 12 h to 16% at 24 h.

### 3.2. C. burnetii Infection Induces Delay in Apoptotic Cell Death in Human Neutrophils

**Figure 4:**
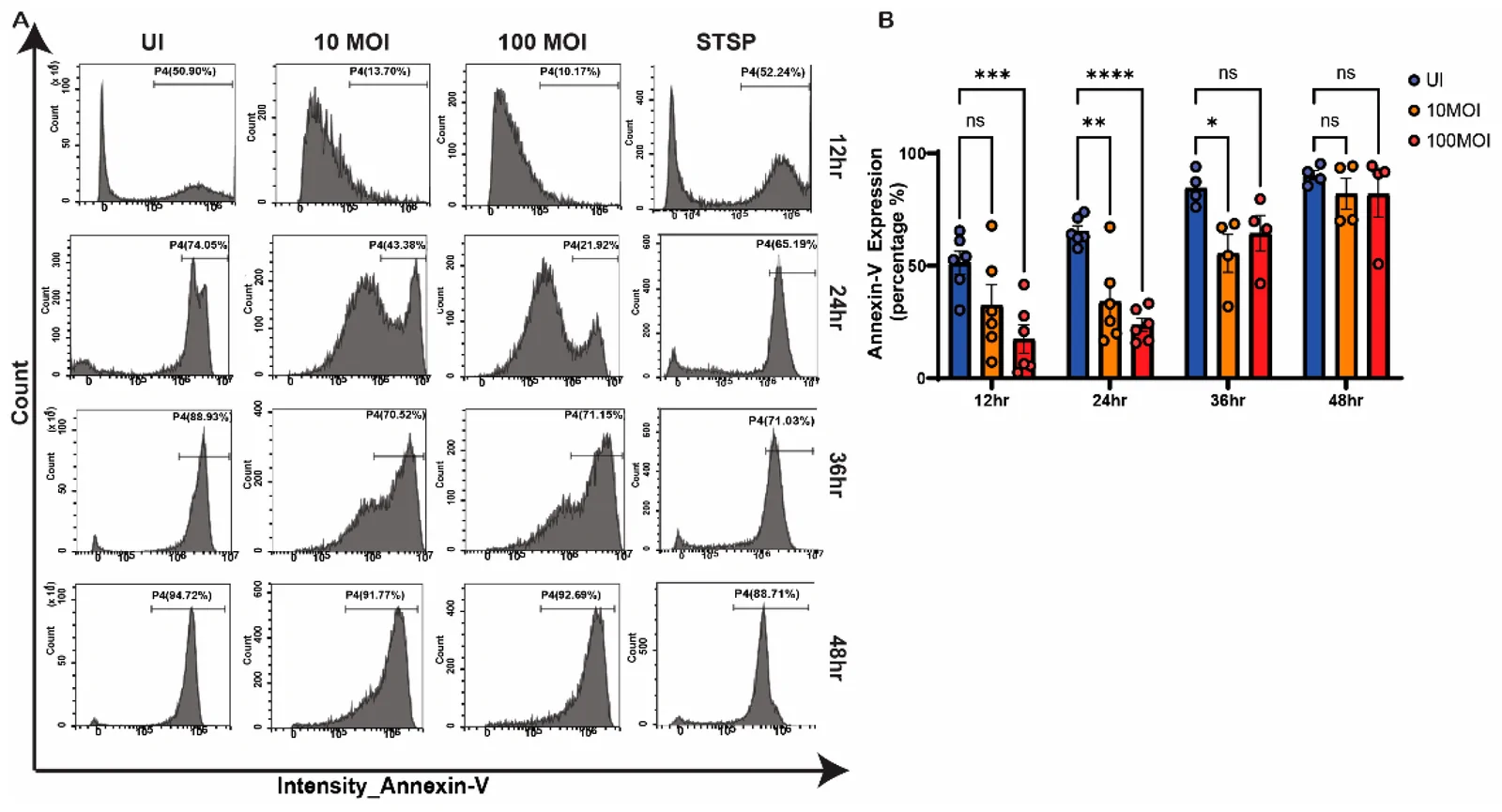
*C. burnetii* Infection causes delay in PS externalization in human neutrophils. (**A**) Apoptosis in uinfected,10 and 100 MOI groups were quantified using Annexin V staining and flow cytometry at 12, 24, 36 and 48 h. Data are mean ± SEM for *n* = 6 (12 and 24 h) and *n* = 4 (36 and 48 h). The samples (UI, 10, and 100 MOI) were processed and analyzed independently for each time point. Annexin-V gates were drawn based on staurosporine (STSP) positive controls specific to each time point at 12, 24, or 36 h. The signal from STSP at these time points was considered the true Annexin-V signal for that particular experiment/time point. (**B**) Graph represents Annexin-V percentage at 12, 24, 36 and 48 h across groups Each dot represents an independent trial. ns non-significant, **** *p* < 0.0001, *** *p* < 0.001, ** *p* < 0.01, * *p* < 0.05 by using ordinary two-way ANOVA, followed by Dunnet’s multiple comparison test.

Isolated human neutrophils were suspended in RPMI supplemented with 10% FBS and infected with *C. burnetii* NMII strain at MOIs of 10 and 100, respectively. The cells were then incubated for up to 48 h to assess the effect of *C. burnetii* on apoptosis by evaluating phosphatidylserine expression via Annexin-V staining and flow cytometry and the activity of caspase 3 at 12, 24, 36, and 48 h. As shown in Figure 4 and Table 1, approximately 51.28% ± 5.1% of uninfected neutrophils displayed Annexin-V staining as early as 12 h post-isolation. In contrast, only 32.51% ± 8.9% and 17.41% ± 6.3% of neutrophils in the 10 and 100 MOI groups, respectively, were positive for Annexin-V. A significant decrease in Annexin-V staining was noted between the uninfected and 100 MOI groups at 12 h (Figure 4B). By 24 h, 82.88% ± 4.3% of uninfected neutrophils were Annexin-V positive, in stark contrast to 34.09% ± 7.6% in the 10 MOI group and 23.61% ± 2.9% in the 100 MOI group. This marked a significant reduction in apoptotic cells in both infected groups compared to the uninfected control. At 36 h, a significant reduction relative to uninfected cells (84.66% ± 3.9%) was observed only in the 10 MOI group (55.50% ± 8.4%), but not in the 100 MOI group (64.42% ± 7.9%). By 48 h, no significant differences were observed among the groups. Interestingly, neutrophils infected with *C. burnetii* showed notably reduced Annexin-V staining levels within the first 24 h. Beyond this period, both infected and uninfected cells exhibited similar Annexin-V staining levels, nearing 95%. These results indicated that the most significant effects occurred at 24 h.

**Figure 5:**
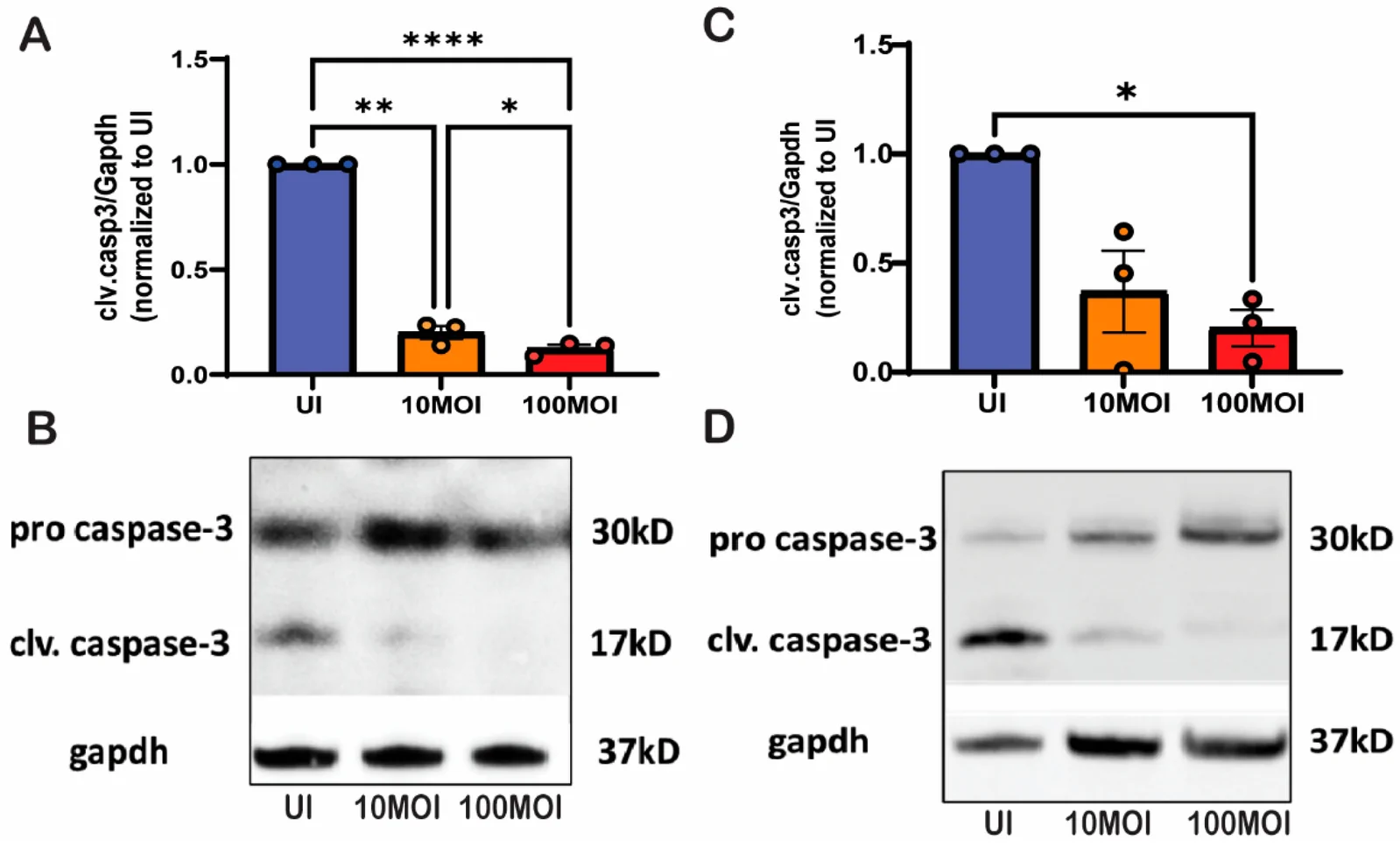
Neutrophils express reduced caspase turnover with *C. burnetii* infection. Expression levels of Caspase-3 and cleaved caspase-3 levels were detected using Western blot. Data are presented for 12 and 24 h as the fold changes with respect to UI. (**B**,**D**) ratio of cleaved caspase-3 to pro caspase-3. (**A**,**C**) Representative blots at 12 and 24 h. **** *p* < 0.0001, ** *p* < 0.01, * *p* < 0.05 by using RM one way ANOVA followed by Tukey’s multiple comparison test.

Caspase-3 is a crucial effector caspase in human neutrophils, pivotal in the proteolytic cleavage of cellular targets leading to apoptotic cell death. It is initially synthesized as an inactive proenzyme and subsequently processed into cleaved caspase-3, forming an active mature enzyme. We employed fluorescent Western blotting followed by densitometric analysis to quantify the turnover of procaspase-3 to cleaved caspase-3 (active form) in both untreated and infected neutrophils, focusing on 12 and 24 h time points. At the 12 h mark, untreated neutrophils undergoing apoptosis showed processing of procaspase-3 to its active form, evidenced by the appearance of a faint cleaved caspase-3 band (17 kDa) as depicted in Figure 5B. This processing was significantly more pronounced at 24 h, indicated by an increase in the intensity of the cleaved caspase-3 band (17 kDa) and a concurrent reduction in the intensity of the procaspase-3 zymogen band (32 kDa), as shown in Figure 5D. In contrast, the processing of procaspase-3 was notably diminished and delayed in both the 10 and 100 MOI-infected groups, demonstrated by denser procaspase-3 bands relative to cleaved caspase-3 at both 12 and 24 h. The density of the bands was measured using iBright software and plotted as the ratio of procaspase-3 band density to cleaved caspase-3 band density, normalized to GAPDH (Figure 5A,C). The ratios were further normalized to the uninfected groups to better represent the fold change in caspase turnover between the infected and uninfected groups.

### 3.3. Phagocytized C. burnetii Activates Autophagy in Human Neutrophils

**Figure 6:**
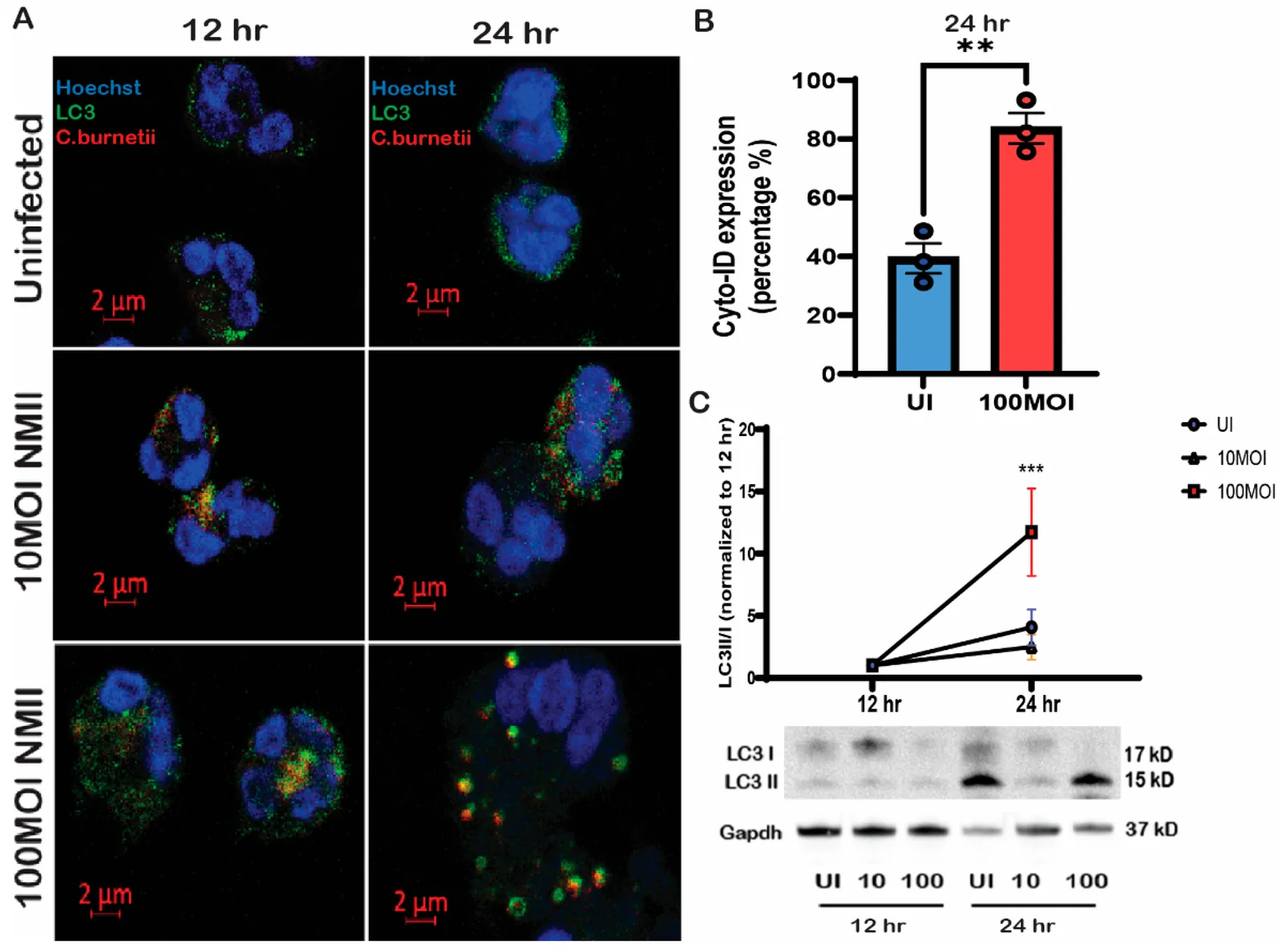
NMII *C. burnetii* induces autophagy at 100 MOI 24 h. (**A**) Representative images of human neutrophils from 10 MOI and 100 MOI groups, at 12 and 24 h, immunostained for DNA (blue), LC3 (green), anti-*Coxiella* (red). Scale bars: 2 µm. (**B**) Bar graph representing the percentage population positive for CYTO-ID in uninfected and 100 MOI-infected group. Data are mean ± SEM for *n* = 3. ** *p* < 0.01 using paired Student’s *t*-test. (**C**) Graph represents fold change in LC3II/I turnover from 12 to 24 h (normalized to GAPDH) in UI, 10 MOI and 100 MOI groups. Each dot represents an independent trial. Representative blots of LC3 I and LC3 II along with loading control GAPDH. *** *p* < 0.001 using RM two-way ANOVA and Sidak’s multiple comparison test.

LC3 plays a crucial role in autophagosome biogenesis and is commonly used as a marker to monitor autophagic activity. Our previous study has shown that NMII bacteria activated LC3-mediated autophagy in bone marrow-derived murine neutrophils [10]. To examine whether *C. burnetii* NMII strain infection could activate autophagy in human neutrophils, we compared the expression of LC3 protein among uninfected, 10, and 100 MOI groups at 12 and 24 h post-infection, respectively. As shown in Figure 6A, qualitative analysis of confocal microscopic images—following immunostaining against *C. burnetii* (red), LC3 (green), and Hoechst (blue)—revealed an increased presence of LC3 puncta in the cytoplasm of the 10 and 100 MOI groups, as well as vacuoles adorned with LC3 in the 100 MOI-infected group at 24 h. For a quantitative analysis of these LC3 vacuoles in the 100 MOI group at 24 h, we utilized CYTO-ID, a cationic amphiphilic tracer dye that efficiently partitions into cells to label vacuoles associated with the autophagy pathway. As shown in Figure 6B, cytometric analysis indicated that 83.63% ± 5.15% of cells infected with 100 MOI *C. burnetii* were positive for CYTO-ID staining. This represents a significant increase compared to the 39.37% ± 5.45% of uninfected cells. Considering that LC3-II not only marks the formation of autophagosomes but is also degraded after lysosomal fusion—thus serving as a transient indicator of autophagic activity; we aimed to differentiate between enhanced autophagosome formation and impaired lysosomal degradation. Since the imaging techniques could not distinguish between LC3-I and LC3-II, we measured the turnover of LC3-I to LC3-II by probing proteins extracted from the uninfected, 10 MOI, and 100 MOI of *C. burnetii* infected groups at 12 and 24 h via Western blotting. As shown in Figure 6C, immunoblotting for the LC3 protein revealed two bands at 17 kDa and 15 kDa, corresponding to LC3-I and LC3-II, respectively. The intensity of these bands was quantified using iBright analysis software. LC3 turnover was calculated by dividing the band intensities of LC3-II by LC3-I after normalization to GAPDH. We observed a significant tenfold increase in LC3 turnover (LC3-II/LC3-I) at 24 h in the 100 MOI group, corroborating our earlier assessments.

### 3.4. C. burnetii-Induced Activation of Autophagy Contributed to Its Ability to Inhibit Apoptosis in Human Neutrophils

**Figure 7:**
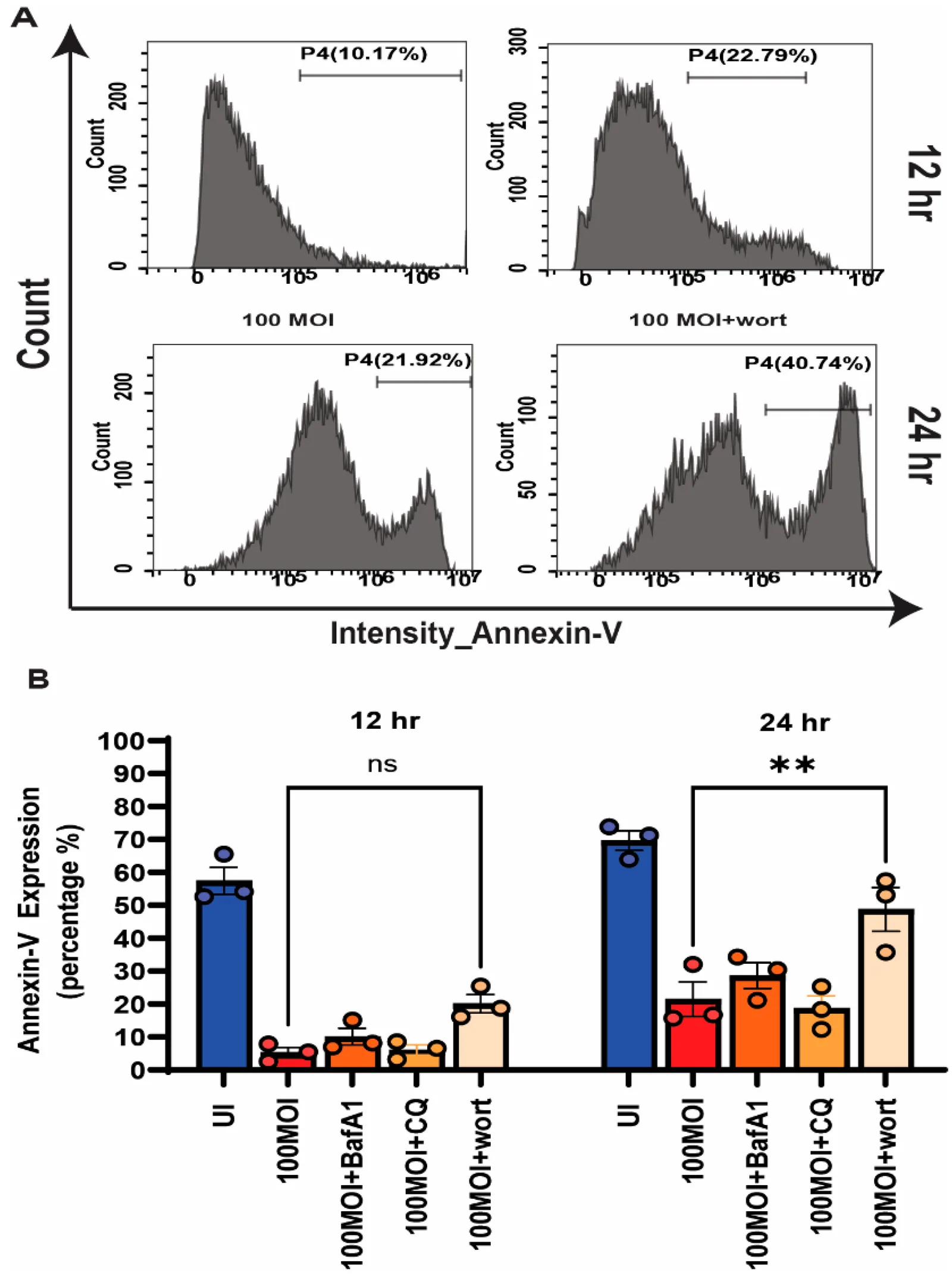
Inhibiting autophagy leads to partial reinstatement of apoptosis in human neutrophils. Apoptosis was quantified using Annexin V staining and flow cytometry. (**A**) Representative plots for 100 MOI and 100 MOI + wortmannin at 12 and 24 h. Annexin-V gates were drawn based on staurosporine (STSP) positive controls specific to each time point at 12 and 24 h. The signal from STSP at these time points was considered the true Annexin-V signal for that particular experiment/time point. (**B**) Graph represents Annexin-V percentage at 12 and 24 h across groups. Data are mean ± SEM for *n* = 3. Each dot represents an independent trial. ns, not significant; ** *p* < 0.01 by RM two-way ANOVA and Tukey’s multiple comparison test.

To investigate the link between *C. burnetii*-induced activation of autophagy and the delayed apoptosis (Figure 4 and Figure 5), we pretreated neutrophils with autophagy inhibitors, including bafilomycin A1 (BafA1; 100 nM), chloroquine (25 µM), and wortmannin (100 nM), before infecting them with a 100 MOI of *C. burnetii*. To assess apoptosis, cells from both uninfected and infected groups were stained with Annexin V at 12 and 24 h post-infection, respectively. As shown in Figure 7, flow cytometric analysis indicated a notable increase in Annexin V expression in the 100 MOI group pretreated with 100 nM wortmannin, showing 20.37% ± 2.8% at 12 h and 48.75% ± 6.6% at 24 h. The increase in apoptotic neutrophils was significantly higher at 24 h compared to the 100 MOI-infected group that did not receive wortmannin treatment (23.83% ± 3.8%). These results suggest that *C. burnetii*-induced activation of autophagy contributed to its ability to inhibit apoptosis in human neutrophils.

### 3.5. C. burnetii Infection Induces Human Neutrophil Death at the Late Stage of Infection

**Figure 8:**
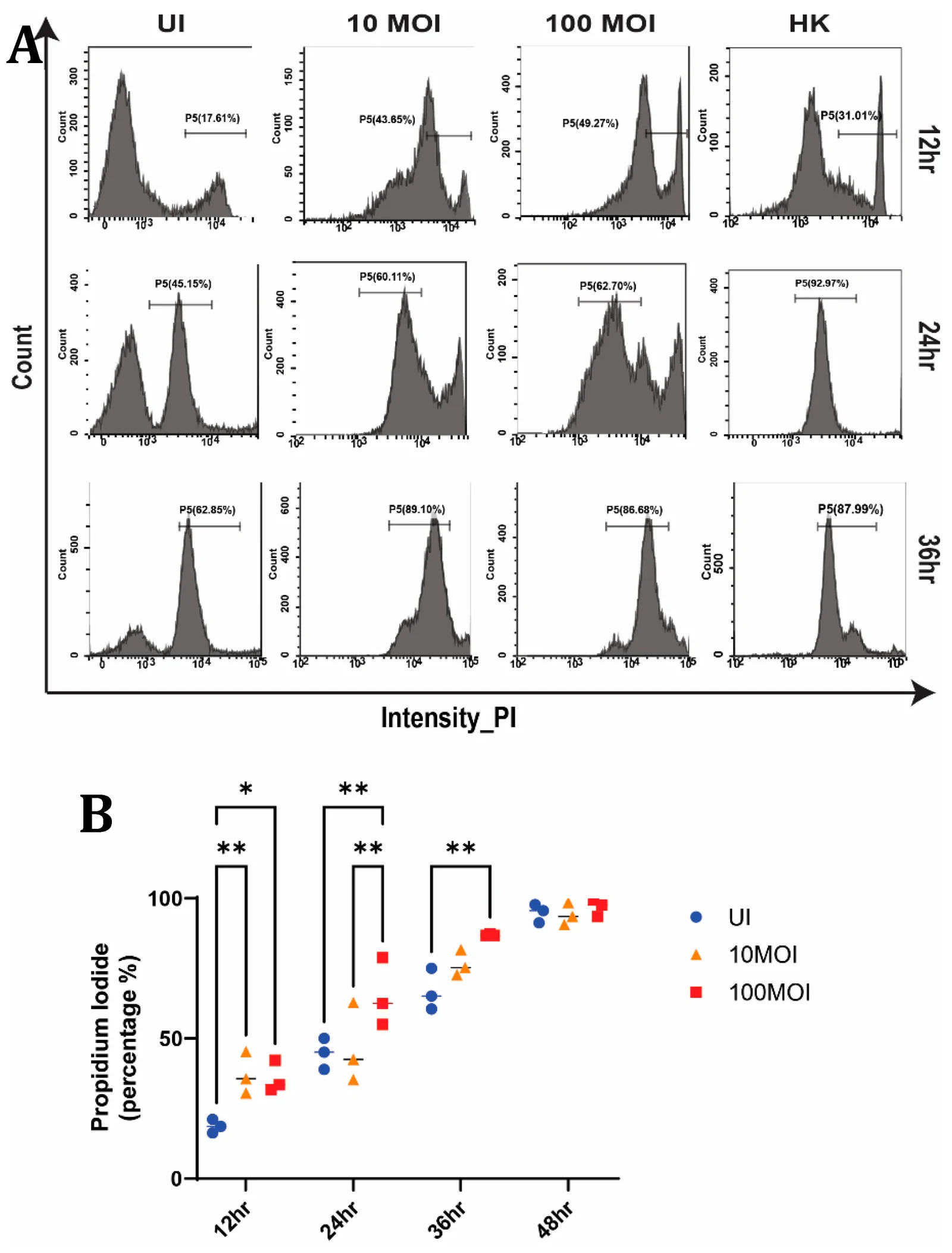
*C. burnetii* induces neutrophil cell death at late stage of infection. (**A**) Panel represents flow cytometry histogram population shift in propidium iodide positive cells in uninfected, 10 MOI, 100 MOI and heat killed (HK, positive control) groups at 12, 24 and 36 h. PI gates were drawn based on individual experimental positive controls. The signal from the heat-killed control samples was considered the true signal, and gating was applied to UI, 10, and 100 MOI samples at each specific time point. (**B**) The bar graph represents data from the three independent experiments. Data are mean ± SEM for *n* = 3. * *p* < 0.05, ** *p* < 0.01 by ordinary two-way ANOVA, followed by Dunnet’s multiple comparison test.

Apoptosis is not the sole cell death mechanism in neutrophils. To determine whether *C. burnetii* inhibiting apoptosis leads to extended neutrophil survival and longevity, we stained *C. burnetii* NMII strain-infected neutrophils with propidium iodide (PI) at 12, 24, and 36 h for flow cytometry analysis. As shown in Figure 8A, uninfected (UI) neutrophils exhibited a gradual increase in PI staining over time, correlating with the rise in annexin expression previously noted in Figure 4. As shown in Table 2, at 12 and 24 h post-infection, the percentage of PI-positive cells was significantly higher in both the 10 MOI group (37.14% ± 4.3% and 46.85% ± 8.2%) and the 100 MOI group (35.79% ± 3.2% and 65.50% ± 7.1%) compared to the uninfected group (18.66% ± 1.3% and 44.72% ± 3.2%). The resulting histogram plots (Figure 8A) illustrate this population shift across the UI, 10, and 100 MOI groups. This experiment was repeated three times, and the compiled data are presented in Figure 8B.

### 3.6. Human Neutrophils Form Lytic DNA NETs to Trap C. burnetii

**Figure 9:**
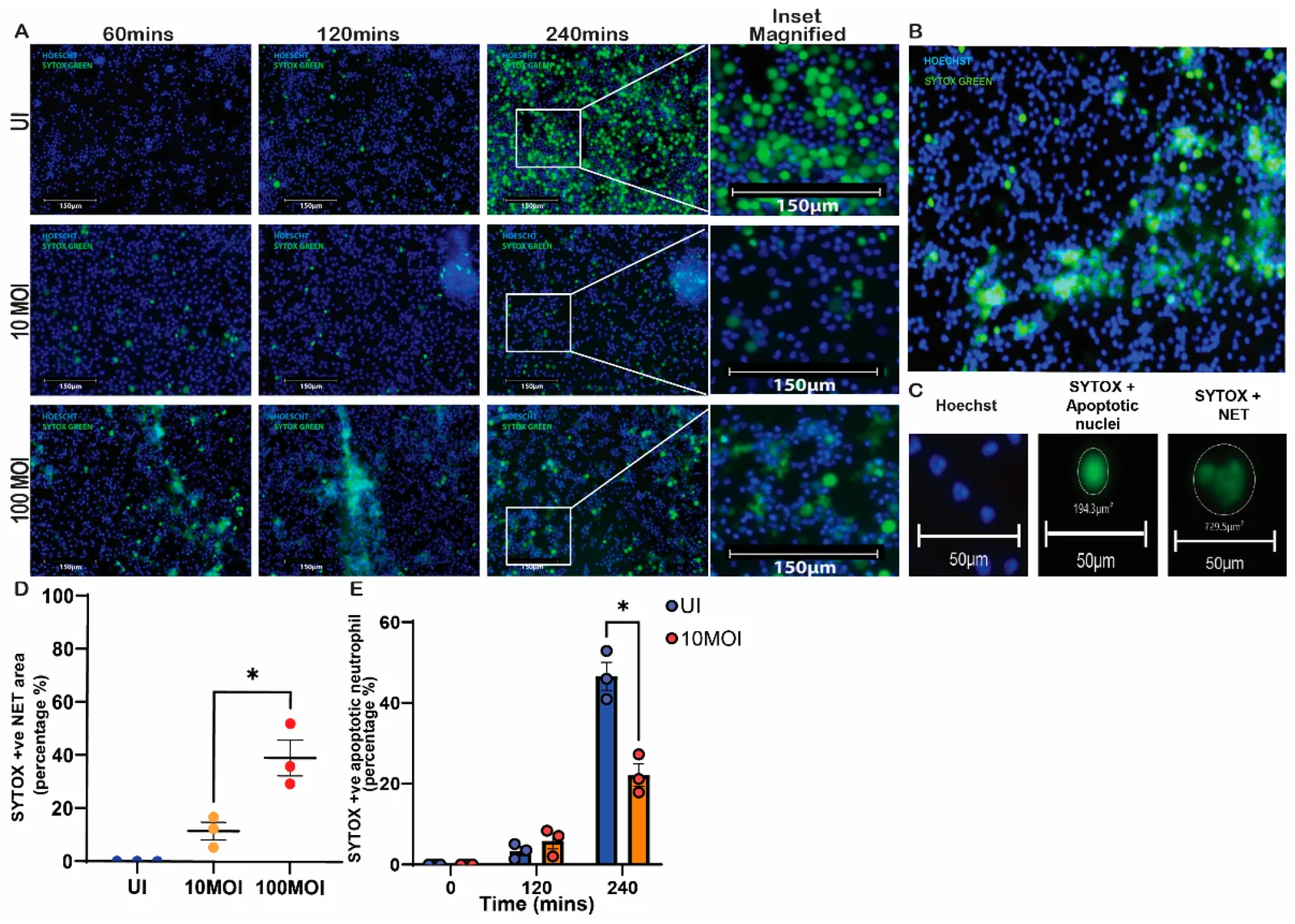
Live-cell imaging to study neutrophil cell death. (**A**) Representative images of human neutrophils from untreated, 10 MOI and 100 MOI groups, at specific time points 60, 120 and 240 min from time lapse video acquired across 240 min. Inset shows magnified images from the panel. Scale bars: 150 µm. (**B**) Representative image of NET like traps formed upon 100 MOI. Scale bars: 150 µm (**C**) Panel showing representative picture of live-cell DNA, SYTOX+ Apoptotic Nuclei and SYTOX+ NET release. Scale bars: 50 µm. (**D**) Data are percentage area covered by NETs at UI, 10 and 100 MOI groups by the end of 240 min. (**E**) Graph represents the SYTOX+ apoptotic nuclei counted from uninfected and 10 MOI conditions. Data from three different experiments. * *p* < 0.05 using RM two-way ANOVA and Sidak’s multiple comparison test.

Live-cell imaging was used to investigate whether human neutrophils could form lytic DNA NETs to trap *C. burnetii*. Neutrophils were incubated with Hoechst (blue) and SYTOX Green (green). After a 30 min incubation, cells were then infected with *C. burnetii* NMII at MOIs of 10 and 100, respectively. As shown in Figure 9A, live microscopy revealed that cells increasingly exhibited SYTOX positivity over time, as illustrated from left to right. Images were captured every 3 min for a total of 4 h, revealing differential responses under each condition. Uninfected neutrophils demonstrated signs of apoptosis (Figure 9A, Row 1), characterized by a symmetrical, circular appearance of the SYTOX stain confined within the cell membrane. In contrast, neutrophils infected with 10 MOI *C. burnetii* displayed swarming and migration into small clusters, followed by localized NET deployment, as depicted in the inset of Row 2 (Figure 9A). In the 100 MOI group, immediate NET formation occurred, entrapping *C. burnetii* and neighboring neutrophils to form extensive mesh-like structures (Figure 9B). We quantified the covered area for each condition using ImageJ software. The findings indicated that the quantified areas were 11.53% ± 3.3% for the 10 MOI and 39.21% ± 6.7% for the 100 MOI (Figure 9D). In addition, as shown in Figure 9C, we also categorized SYTOX-positive cells as either SYTOX+ apoptotic cells or SYTOX+ NETs based on their morphology. Our observations indicated that while the uninfected control cells primarily exhibited SYTOX+ apoptotic nuclei, the 10 MOI presented a mix of both types. To assess the impact of *C. burnetii* on bystander, non-NET-releasing neutrophils, we compared the percentage of SYTOX+ apoptotic cells in the 10-MOI-infected group against the uninfected control (Figure 9E). This analysis revealed that the 10-MOI-infected cells exhibited a significantly lower proportion of apoptotic cells (22.11% ± 2.70%) compared to the uninfected control (46.58% ± 3.45%). These results provided further evidence to support that *C. burnetii* NMII infection can inhibit apoptosis in human neutrophils at the early stage of infection.

### 3.7. MPO and ROS Are the Main Effectors in C. burnetii-Induced Human Neutrophil NETosis

**Figure 10:**
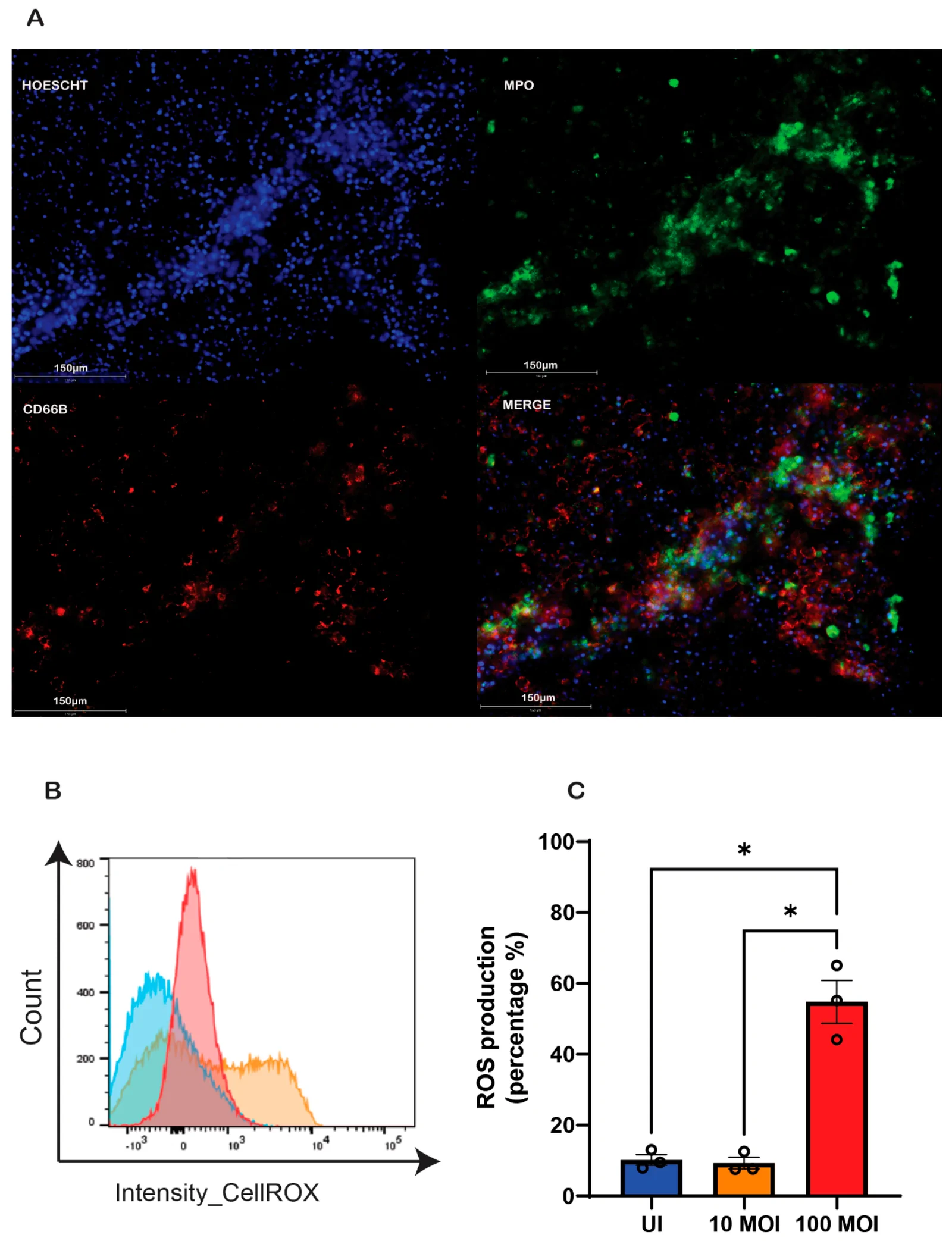
MPO and ROS are the main effectors in NETosis. (**A**) Panel represents DNA (cyan), MPO (green) and CD66b (red) and merge. Scale bars: 150 µm. (**B**) FACS histogram of CELLROX staining showing a shift in 100 MOI group. UI (blue), 10MOI (orange), 100 MOI (Red) (**C**) Graph represents the ROS percentage from three different experiments. * *p* < 0.05 using RM one-way ANOVA and Tukey’s multiple comparison Test.

To further characterize lytic cell death upon *C. burnetii* infection, markers of necrotic cell death such as reactive oxygen species (ROS) and myeloperoxidase (MPO) were measured post-live-cell imaging employing flow cytometry and immunostaining techniques. As shown in Figure 10B, the production of ROS in these cells was quantified using CellROX and flow cytometry. We found that concurrent with the increase in NET formation, the 100 MOI group exhibited a marked rise in ROS production, reaching 54.7% ± 6.0% (Figure 10C). It is important to note that MPO (Figure 10A) is only one of several markers indicative of NET formation in the current study. For a more comprehensive characterization, further analyses using markers such as citrullinated histone H3 and neutrophil elastase are necessary.

## 4. Discussion

*C. burnetii* is an obligate intracellular pathogen that causes acute and chronic Q fever in humans. Its pathogenicity is attributed to its ability to circumvent innate immune defenses and invade immune cells, including macrophages and neutrophils [10,19]. Neutrophils are primary responders to bacterial infections. They typically exhibit a short lifespan, undergoing spontaneous apoptosis approximately 24 h post-release into circulation [20]. This precisely controlled lifespan is closely linked to cellular functionality, as disruptions in neutrophil turnover can lead to aggravated disease and tissue damage [21]. Our previous study indicated that *C. burnetii* infection substantially prolongs neutrophil lifespan in mice [22]; however, its impact on human neutrophils has yet to be investigated.

In this study, we explored whether the *C. burnetii* NMII strain can influence apoptosis in human neutrophils. Following established protocols [23], we evaluated the rate and degree of apoptosis and cell death in human neutrophils in vitro. Neutrophils exhibiting phosphatidylserine (PS) externalization are marked for macrophage phagocytosis through a process called efferocytosis [24], which is required to prevent neutrophil accumulation and subsequent inflammation. Our findings, shown in Figure 4, indicated that approximately half of the untreated control neutrophils began to exhibit signs of apoptosis between 6 and 12 h, which increased to nearly all cells by the 24 h mark. In contrast, a significantly lower percentage of human neutrophils infected with *C. burnetii* showed signs of apoptosis, as assessed by measuring PS externalization using an Annexin V conjugate and flow cytometry. Notably, the reduction in PS externalization in infected cells indicated a delay rather than an outright inhibition, as these cells eventually underwent apoptosis between 24 and 48 h. A similar delay in neutrophil PS externalization has been demonstrated by another major intracellular pathogen, *F. tularensis* [25].

Additionally, we observed an inhibition of procaspase-3 cleavage into effector caspase-3 in human neutrophils infected with *C. burnetii*, further substantiating our hypothesis (Figure 5). Caspase-3 activation is crucial to the neutrophil apoptotic process. A previous study from our laboratory demonstrated that *C. burnetii* NMII infection inhibits cleaved caspase-3 in mouse neutrophils [22]. Furthermore, prior research has shown that human neutrophils infected with *Leishmania major* experience a suppression of caspase-3 activation [26], and *Chlamydia pneumoniae* infections have been associated with delayed neutrophil apoptosis due to decreased caspase-3 activity [27]. Together, these findings imply that the NMII strain’s capacity to delay apoptosis in neutrophils is mechanistically linked to its ability to suppress caspase-3 activity, consequently prolonging the lifespan of these cells.

Autophagy, a cellular process essential for the degradation and recycling of cellular components, plays a crucial role in regulating apoptosis, particularly in neutrophils [28]. To explore the influence of *C. burnetii* infection on neutrophil autophagy, our study quantified LC3 turnover in human neutrophils. We found that neutrophils infected with *C. burnetii* NMII, particularly at 100 MOI, exhibited a significant increase in LC3 II turnover (Figure 6), indicating an induction of the autophagic process. This aligns with previous studies in our laboratory, which observed similar increases in NMII-infected mouse neutrophils [9]. However, the specific pathways through which autophagy contributes to apoptosis inhibition remain unclear. Although LC3 turnover is the gold standard for monitoring autophagy, increased turnover can signify either an induction of autophagy or an interruption of the autophagic flux [28]. To address this, we utilized inhibitors targeting various stages of autophagy. Our observations revealed that bafilomycin and chloroquine, which inhibit the fusion of autophagosomes and lysosomes toward the end of the autophagic process, did not affect the delayed apoptosis caused by *C. burnetii*. In contrast, treatment with wortmannin, an inhibitor of class III PI3K involved in autophagy, partially restored apoptosis in infected neutrophils (Figure 7). Previous findings from our laboratory show that *C. burnetii* NMII inhibits apoptosis by activating the PI3K survival pathway and induces the expression or stabilization of the anti-apoptotic protein Mcl-1 [22]. Similar mechanisms involving PI3K in apoptosis inhibition have been noted in other intracellular pathogens [29]. However, it has been primarily class I PI3Kα and Akt-mediated PI3K pathways that have been implicated in the survival of neutrophils, leaving room for further investigation into these complex molecular interactions.

In this study, we also demonstrate that neutrophils undergo extensive cell death when infected with 10 and 100 MOI NMII *C. burnetii*, as shown in Figure 8. To delve deeper and identify the specific type of cell death, we employed live-cell imaging techniques using Hoechst, a cell-permeable DNA stain, and SYTOX Green, a cell-impermeable DNA stain [30]. Within an hour, cells from the uninfected, 10 MOI, and 100 MOI groups exhibited positive staining for SYTOX Green, indicating compromised membrane integrity (Figure 9A). Upon detailed examination, it was evident that uninfected neutrophils were undergoing constitutive apoptosis, with the stained DNA remaining within the cell membrane. However, the groups infected with *C. burnetii* presented a different scenario. These infected cells expelled DNA extracellularly, forming structures morphologically resembling Neutrophil Extracellular Traps (NETs). The nature of these NET-like structures varied significantly between 10 MOI and 100 MOI. At 10 MOI, neutrophils formed localized, smaller NETs and recruited nearby neutrophils to form local clusters (Figure 9A). In contrast, 100 MOI triggered extensive recruitment of neutrophils and the formation of large-scale NETs (Figure 9A). The areas covered by these NET-like structures at 10 and 100 MOI are quantified in Figure 9D.

In addition to the observations on NET-like formation, our study noted that neutrophils that did not form NETs, but were infected with 10 MOI *C. burnetii*, exhibited a delay in apoptosis compared to the uninfected group, as quantified in Figure 9E. This could not be verified in the 100 MOI group due to comparatively higher swarming and lytic cell death, which pose significant challenges in accurately characterizing and quantifying apoptotic neutrophils alone. This delayed apoptosis in non-NET-forming, infected neutrophils underscores the diverse impact of *C. burnetii* infection on neutrophil fate and function, highlighting the pathogen’s capability to modulate host cell death pathways through multiple mechanisms. Following live-cell imaging, we conducted further analyses to characterize the cell death pathways. We measured the levels of ROS using flow cytometry and detected MPO through immunostaining. Notably, there was a significant increase in ROS in the 100 MOI group, which, when coupled with the observed MPO staining on necrotic cells (Figure 10), partially confirms the occurrence of NET formation.

Previous studies have provided compelling examples of neutrophils undergoing necrotic cell death following bacterial infection. One notable instance involved *Mycobacterium tuberculosis* evading the antibacterial actions of neutrophils and macrophages. It was observed that ROS-mediated necrosis of neutrophils, triggered by *M. tuberculosis*, played a vital role in allowing the pathogen to escape macrophage-mediated clearance. This evasion strategy enabled *M. tuberculosis* to replicate and induce necrosis in subsequent host cells [31]. Interestingly, when MPO activity in infected neutrophils was inhibited, necrosis was blocked, thereby restoring macrophage control over mycobacterial growth. Another example is found in the context of pneumococcal infections. Components of the *pneumococcus*, such as pneumolysin, hydrogen peroxide, and cell wall elements, have been shown to initiate inflammation and suppress host defenses. The induction of necrosis in neutrophils by viable pneumococci was found to aid the spread of bacterial infection [32]. Conversely, metabolically inactive bacteria induced neutrophil apoptosis, which was likely followed by macrophage phagocytosis, helping to resolve the inflammation.

The intricate dynamics between neutrophil defensive mechanisms and pathogen strategies, as illustrated in these examples, underscore the necessity for more in-depth research. Particularly, further experiments are needed to assess the viability of *C. burnetii* and precisely identify the type of necrotic cell death occurring in neutrophils. This is especially relevant for determining whether the observed cell death strictly aligns with NETosis, a specific form of regulated necrosis.

The phenomenon observed in our study might be explained by previous findings showing that *C. burnetii* can inhibit the NADPH oxidase-mediated respiratory burst in human neutrophils [11]. This inhibition could disrupt autophagy-driven programmed cell death (PICD), leading neutrophils to undergo necrotic cell death instead. This hypothesis is supported by our observations in Figure 3, where we noted the presence of MPO and *C. burnetii* within 100 MOI-infected neutrophils. However, there was no co-localization, further suggesting *C. burnetii*’s ability to evade the intracellular neutrophil response. In contrast, during necrotic cell death, the probability of *C. burnetii* being exposed to extracellular MPO might increase substantially (Figure 10A).

Interestingly, our findings also highlight a key difference between murine and human neutrophils. When the phagocytic elimination of pathogens is hindered, as is the case with *C. burnetii* infection, human neutrophils appear to switch to a more severe type of inflammatory cell death. This observation leads us to hypothesize that human neutrophils form NETs against *C. burnetii* as an effective countermeasure to the pathogen’s intracellular dominance. The formation of NETs could represent a crucial adaptive response, enabling neutrophils to combat pathogens that have evolved mechanisms to evade traditional phagocytic killing. This hypothesis underscores the dynamic and adaptable nature of neutrophil responses in human immunity and highlights potential differences in immune response mechanisms between species.

## 5. Conclusions

This study highlights that human neutrophils, unlike their mouse counterparts, may adopt more efficient strategies to combat *C. burnetii*, such as forming extensive neutrophil extracellular traps (NETs). While *C. burnetii* can prolong neutrophil lifespan and inhibit apoptosis by suppressing caspase-3 activation, human neutrophils exhibit an enhanced lytic response. At high multiplicities of infection, NET formation becomes a crucial mechanism, counteracting the pathogen’s evasion tactics. This suggests that human neutrophils, through NETosis, may provide a stronger defense against *C. burnetii*, representing a distinct and potentially superior immune strategy.

## Figures and Tables

**Table 1:** Annexin-V expression in human neutrophils infected with *C. burnetii*.

Treatment/ Time (h)	Uninfected (Mean% ± SEM%)	10 MOI (Mean% ± SEM%)	100 MOI (Mean% ± SEM%)	Trials (n)
12	51.28 ± 5.2	32.51 ± 8.9	17.41 ± 6.3	6
24	65.32 ± 2.5	34.09 ± 7.6	23.61 ± 2.9	6
36	84.66 ± 3.9	55.50 ± 8.4	64.42 ± 7.9	4
48	90.25 ± 2.1	82.04 ± 6.8	81.94 ± 10.4	4

**Table 2:** Propidium iodide expression in human neutrophils infected with *C. burnetii*.

Treatment/ Time (h)	Uninfected (Mean% ± SEM%)	10 MOI (Mean% ± SEM%)	100 MOI (Mean% ± SEM%)	Trials (n)
12	18.66 ± 1.3	37.14 ± 4.3	35.79 ± 3.2	3
24	44.72 ± 3.2	46.85 ± 8.2	65.50 ± 7.1	3
36	66.86 ± 4.2	76.57 ± 2.6	86.99 ± 0.2	3

## Data Availability

All data are comprised within this manuscript.

## Abbreviations

PS	Phosphatidylserine
NETs	Neutrophil Extracellular Traps
MPO	Myeloperoxidase
ROS	Reactive Oxygen Species
NADPH	Nicotinamide Adenine Dinucleotide Phosphate Hydrogen
ARDS	Acute respiratory distress syndrome
EDTA	Ethylenediaminetetraacetic Acid
MOI	Multiplicity of Infection
PICD	Pathogen-Induced Cell Death
